# Supplementary material for: Resource competition and coexistence in heterogeneous metacommunities: many-species coexistence is unlikely to be facilitated by spatial variation in resources
Source: PeerJ. 2013 Aug 22;1:e136. doi: 10.7717/peerj.136 (PMC3757524; doi:10.7717/peerj.136)
Supplement: Supplemental Appendix S1 — Derivation of standardized density vjx. [file peerj-01-136-s001.docx]

**Supplemental appendix S1: Derivation of standardized densities, *v_jx_***

The second-order approximation of the low-density metacommunity-scale growth rate of an invader requires first-order approximations of the relative densities of the resident and invader, *v_jx_*. For ease of calculation, I standardize the relative densities to have a mean of zero, e.g. . Those expressions are derived here.

*Derivation of u_rx_*

The standardized density of the resident is defined as . By definition, the resident density is at equilibrium, . Thus,

.

Eqn. A.1 states that the standardized density of the resident in patch *x* is equal to the standardized supply rate in that patch.

*Derivation of u_ix_*

The standardized density of the invader is . Unfortunately, the general expression for is unknown. My strategy is to estimate *u_ix_(t)* by finding an expression for, then solve this expression for the equilibrium values,. This method of finding *u_ix_(t)* assumes that the standardized relative density of the invader reaches its equilibrium much faster than invader’s density.

The rate of change of the invader is in patch *x* is

.

This can be written in terms of standardized densities and supply rates, , to give

.

A first-order Taylor expansion around , after combining terms, results in

.

Taking a spatial mean of both sides gives

.

By definition, . Taking the time derivative of each side, applying the quotient rule to the right hand side and simplifying gives

,

which yields the equilibrium solution

.
