# Supplementary material for: Resource competition and coexistence in heterogeneous metacommunities: many-species coexistence is unlikely to be facilitated by spatial variation in resources
Source: PeerJ. 2013 Aug 22;1:e136. doi: 10.7717/peerj.136 (PMC3757524; doi:10.7717/peerj.136)
Supplement: Supplemental Appendix S2 — Simulation of Many-Species Dynamics. [file peerj-01-136-s002.docx]

**Supplemental appendix S2. Simulation of Dynamics**

This appendix presents the results of simulations of the dynamics of 50 species in 20 patches to demonstrate coexistence of only two species is possible for both linear and saturating resource dependent growth functions and for large variation in resource supply rates.

For the simulations, supply rates were selected at random from a log-normal distribution ($\mu$=1, $\sigma$=1.5). Species specific parameters were assigned so that there is a linear tradeoff between dispersal and competitive ability, such that species that retain a higher proportion of offspring in the natal patch (higher *p*) have higher *R^*^*. For the case of linear growth functions, eqns 3 from the main text were simulated with *m*=.2, *Q*=0.5 for all species, *p_i_*=*i/n_sp_* and *b_i_*=*m_i_/(1+p_i_),* number of species, *n_sp_* = 50 (Figure B2).

Figure B1 shows that at equilibrium, all but two species go extinct (represented by blue and green lines). The surviving species are those that occupy the ends of the tradeoff spectrum shown in Figure B2 as large points. The green line represents a species that experiences the least interpatch dispersal and has the highest *R^*^*. The blue line represents the species that experiences the most interpatch dispersal and has the lowest *R^*^*. These results are consistent with the invasion analysis presented in the Figure 2, which shows that a system of patches inhabited by two resident species, one at either end of the tradeoff spectrum is uninvasible by a species with an intermediate strategy.

Figure B3 shows simulations of dynamics of species whose growth is a saturating function of resource concentration and resources are lost at a proportional rate *aR_x_*, resulting in the system

$\begin{matrix} \frac{dn_{jx}}{dt}=-m_{j}n_{jx}+\mu_{i}(R_{x})\left(p_{j}n_{jx}+(1-p_{j})\left\langle n_{j}\right\rangle_{x}\right) \\ \frac{dR_{x}}{dt}=S_{x}-aR_{x}-\sum_{j} Q_{j}\mu_{j}(R_{x})\left(p_{j}n_{jx}+(1-p_{j})\left\langle n_{j}\right\rangle_{x}\right), \\ \mu_{i}(R)=\frac{\mu_{\max}R}{k_{i}+R}. \end{matrix}$ (B.1)

For these simulations, *a=1*, *m*=4, *Q*=0.5, *μ_max_*=2, *p_i_*=i/n_sp_, $k_{i}=\frac{1}{2}(1+p_{i})(m+\mu_{\max})$. This again results in a linear tradeoff between dispersal and *R^*^*(Figure B4). As with the linear growth response, only two species coexist at equilibrium. Those that coexist inhabit either end of the tradeoff spectrum. No further coexistence was possible by allowing non-linear tradeoffs between dispersal and competition.


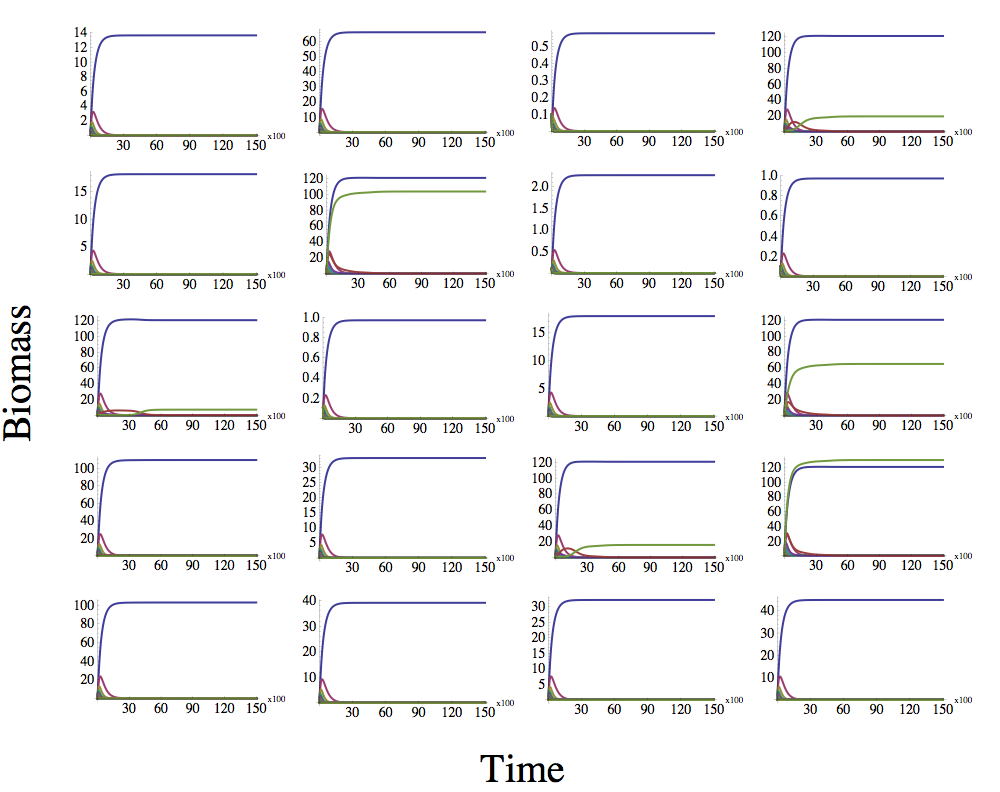


Figure B1. Simulation of Dynamics of eqns 3 for 50 species in 20 patches. Species experience a linear tradeoff between dispersal (*p_i_*) and competitive ability (*R^*^*).


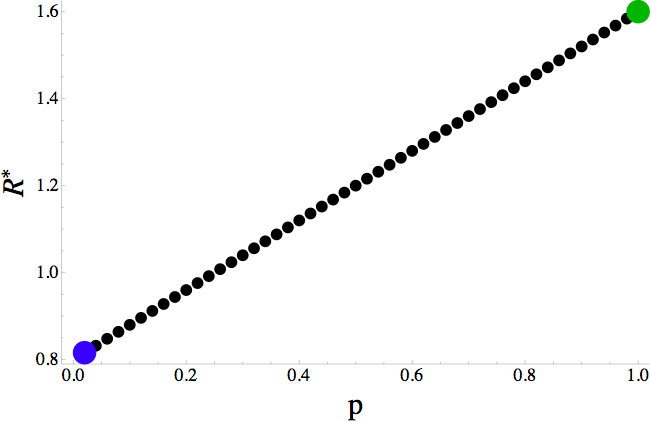


Figure B2. Tradeoff manifold for species in simulations. Large colored dots at either end show position of species that coexist at equilibrium.


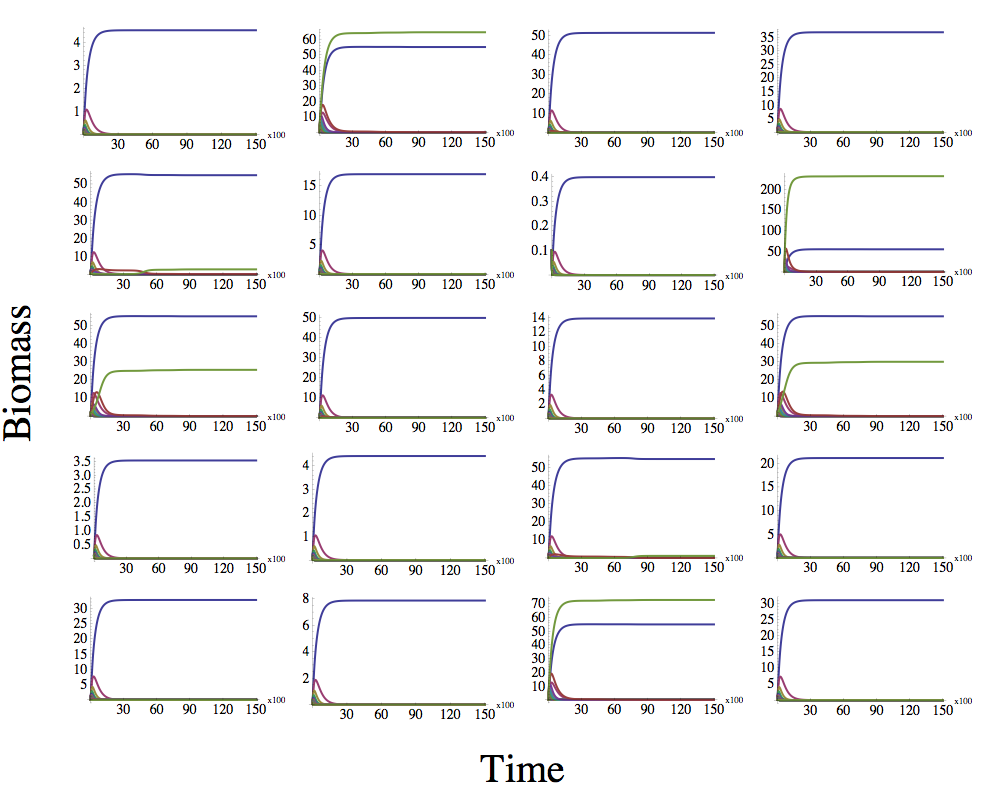


Figure B3. Simulation of Dynamics of eqns. B.1 for 50 species in 20 patches. Species experience a linear tradeoff between dispersal (*p_i_*) and competitive ability (*R^*^*).
